# How Ideas Come Into Being: Tracing Intertextual Moments in Grades of Objectification and Publicness

**DOI:** 10.3389/fpsyg.2019.02355

**Published:** 2019-11-01

**Authors:** Andrea Karsten, Marie-Cécile Bertau

**Affiliations:** ^1^Writing Center, Department of Psychology, Paderborn University, Paderborn, Germany; ^2^Department of Psychology, University of West Georgia, Carrollton, GA, United States

**Keywords:** idea formation, language activity, objectification, intrapersonal intertextuality, articulation, Jakubinskij, Vygotsky, Humboldt

## Abstract

How do ideas come into being? Our contribution takes its starting point in an observation we made in empirical data from a prior study. The data center around an instant of an academic writer’s thinking during the revision of a scientific paper. Through a detailed discourse-oriented micro-analysis, we zoom in on the writer’s thinking activity and uncover the genesis of a complex idea through a sequence of interrelated moments. These moments feature different degrees of “crystallization” of the idea; from gestures, a sketch, a short written note, oral explanations to a final spelled-out written argument. For this contribution, we re-analyze the material, asking how the idea gets formed during the thinking process and how it reaches a tangible form, which is understandable both for the thinker and for other persons. We root our analysis in a notion of language as social, embodied, and dialogical activity, drawing on concepts from Humboldt, Jakubinskij, and Vygotsky. We focus our analysis on three conceptual nodes. The first node is the ebbing and advancing of language in idea formation – observable as a trajectory through linguistically more condensed or more expanded utterance forms. The second node is the degree of objectification that the idea reaches when it is performed differently in a variety of addressivity constellations, i.e., whether and how it becomes understandable to the thinker and to others in the social sphere. Finally, the third node is the saturation of the idea through what we call intrapersonal intertextuality, i.e., its complex and dialogically related re-articulations in a sequence of formative moments. With these considerations, we articulate a clear consequence for theorizing thinking. We hold that thinking is social, embodied, and dialogically organized *because* it is entangled with language. Ideas come into being and become understandable and communicable to other persons only by and within their different, yet, intertextually related formations.

## Introduction

This contribution addresses the intriguing question of how ideas “come into being.” That is, we ask how ideas get formed in thinking processes and how they reach a tangible form, which is communicable to others in the social sphere. Speaking of a communicable public form, we imply that *on their way to* reaching an exterior, socially understandable form, ideas may already crystallize to not-yet-communicable and non-public forms, i.e., forms for the thinker herself or himself. As we intend to show, these forms are different in stability, in their degree of verbal articulation, and in meaning from the final other-addressed form. At the same time, we conceive of both the communicable public forms and the not-yet public forms as dialogical and addressed idea formations, which others and the thinker can perceive and experience due to their embodied and at least partly verbal form, oral or written. Our aim is to formulate a conception of thinking as social, dialogical, and embodied by promoting understanding of language as activity and thinking as language-related process.

We relate our conceptualization of this process to Vygotsky’s (1987) considerations of the dynamics between thinking and speaking where the process of “inner speech” has an explicit formative and mediational role. On these grounds, we are mostly interested in what seems to be a succession of formative moments, a trajectory leading through and connecting different types of forms to each other into one “arch of thinking.” Tracing these formative moments, we propose a close look into the intricate, highly time-sensitive entanglement of thinking and speaking, a movement between these two distinct, yet, inextricably related phenomena.

Observing empirical data from a prior study (Karsten, 2014a,b), we noticed different *degrees in the crystallization* of an idea within the thinking and articulating process of an expert academic writer revising a scientific article. These degrees are coupled with various forms of tangible (re)presentations, reaching from just body movements to pencil drawings on paper with handwriting, and to computer writing. Moreover, these tangible forms vary also in the degree to which linguistic features are used at all, and then how elaborated this usage shows to be: just a few words, or an elaborated sentence that is obviously fitting all the norms of a written piece for the writer’s academic community.

This observation touches on the general question of how language is related to thinking. This issue is usually addressed by excluding language from the formation of thought, since language is traditionally not attributed any formative but only a transmitting function toward thoughts. Therefore, research investigates either idea formation with no mention of language or language production occurring after conceptual formation. For instance, creativity research in the fields of organizational and social psychology considers idea formation on the individual and group level, where discussions and other forms of verbalization appear as secondary products (Paulus, 2000; McAdam and McClelland, 2002; Kohn et al., 2019). Also, cognitive psycholinguistics is still based on a modular notion of speech production with verbal elements as outcomes. Models of oral speech production (Levelt, 1989; Levelt et al., 1999; Pickering and Garrod, 2004) and of the writing process (Hayes and Flower, 1980; Hayes, 1996; Kellogg, 1996) consider language formulations as expressions of formed thoughts downstream from a pre-linguistic level of thinking. However, the notion of articulation we use implies a formative function of language on thinking.

In order to illuminate the process of idea formation in various degrees of crystallization, we re-analyze some core moments of our empirical material. Proposing an alternative theoretical framework for idea formation, we root this analysis in Vygotsky’s notion of semiotic mediation, Humboldt’s understanding of language as activity, and a general process ontological view (section “Theoretical Framework”). Prioritizing processes over substance, we argue that language can be understood as medium in the sense of element, within which individuals form their activities to each other and to themselves in a sequence of interrelated embodied movements. These movements lead to observable constellations of other-addressed and self-addressed utterances in time and space through which ideas come into being.

Setting the ground for the analysis, we present five successive moments from our data, during which our study participant develops an idea that is central to his writing process (section “Studying Idea Formation”). We trace his activities toward clarifying the idea for himself and articulating it for his reader, illustrating the formative sequence with figures and descriptions of the single steps he takes.

In a first analytical move, we connect our data to the work of the Russian dialogist Lev P. Jakubinskij, whose work was seminal for Vygotsky’s conceptualization of the relation between thinking and speech (section “Observing Condensed and Expanded Language Forms”). Jakubinskij (1979) and Yakubinsky (1997) notices that in each communicative activity, there is an oscillation between the verbal and the non-verbal, a movement of more-or-less that is specific to what he calls functional forms of language and their genres (Bertau, 2008). We present a schema of two continua elaborated from Jakubinskij’s observations according to which language forms can be classified, and apply it to our data. As a result, and in line with Jakubinskij’s assumptions, we can confirm our first impression of a movement between ebbing and advancing language forms in our study participant’s idea formation. We also identify moments of seemingly inward-directed activity that cannot be grasped with Jakubinskij’s schema easily. We formulate thus the need to connect Jakubinskij’s observations of the “outer” social phenomenon of other-addressed talk or writing with the phenomenon of inner speech according to Vygotsky (1987), which we reformulate as the spectrum of self-addressed forms of speaking.

A second analytical step focuses on the constellations of who addresses whom in the different sequences in our material and how these addressivity constellations co-influence the respective language forms that can be found (section “Varying Grades of Objectification Depending on the Constellation of Addressivity”). We will discuss how the addressivity constellations are related to certain grades of objectification or publicness and what this implies for the process of our study participant’s idea formation. By objectification, we mean a genuinely language-based process that involves generating a *language-object* as recognizable, delineated entity and that leads to and is tied into *objectivity*, pertaining to common, social, or trans-individual language activity types – in this sense, objectivity amounts to publicness: the forms are fully public because they adhere to the form-and-meaning norms expected by the language community for a certain genre, so that their display will be accepted as “right.”

Our last analytical step highlights the intertextual relations between the various forms we observe and discuss (section “Intrapersonal Intertextuality: A Crucial Process in Idea Formation”). From these observations, we derive that intrapersonal intertextuality, i.e., the movement of interrelated language forms through a series of moments and addressivity constellations while staying always tied back to the speaker-thinker, is crucial for idea formation. It is only through the intertextual saturation of the idea – because it gets articulated and re-articulated to different moments, in different forms and for different addressees – that idea formation is completed and results in an objectified form communicable to social others.

Through our theoretically underpinned analysis, we aim to articulate a clear consequence for theorizing thinking. We hold that thinking is social and dialogically organized *because* it is entangled with language. It is therefore related to others in the social sphere, and it is embodied because it needs and takes the language forms showing different degrees of articulation, i.e., formal (syntax, lexicon, textual coherence) and semantic clarity, that render the forms understandable and communicable to social others. Our interpretation of Vygotsky’s framework of semiotic mediation and the role of inner speech for thinking is at the core of this argument.

## Theoretical Framework

The standard metaphor of thought formation is that of expressing pre-verbal ideas by giving them a verbal form. Within this metaphor, there is an implicit conception of *language as an envelope* ready for the transmission of ideas: thoughts that are already completed get stuck in pre-formed verbal molds and then are given to others to unpack. This leads into representationalism with the basic idea of transfer of the represented cognitive items (Reddy, 1979; Linell, 2009). Many influential models for cognitive and communicative processes in psychology and language philosophy rely on this envelope metaphor.

Vygotsky’s notion of semiotic mediation contrasts this view and conceptualizes *language as a medium* for thinking. The Vygotskian notion of medium is widely interpreted in terms of *language as a tool*, a discrete middle between the thinker and the social world. This is done so by Vygotsky himself in his older writings (e.g., Vygotsky and Luria, 1994, c.f., Keiler, 2002) and by most authors who build on Vygotsky’s work. In this view, people can use the tool of language that society provides to them, but they also can to put it away when the thought is done.

Arguing for a conception of thinking as social, dialogical, and embodied leads us to specify the Vygotskian concept of semiotic mediation in terms of a notion of language that is radically performative and immanent, i.e., not abstractable from its sites and ways of occurring. Performativity and immanence of language as central notions to our argument can also be grasped with the metaphor of *language as medium*. However, we understand the medium precisely not as tool. Rather, the picture we propose is that of *medium as an element*, where the element is a living element allowing for specific lifeforms and activities – as water allows fish to swim. However, language as element is not pre-existing language activity as water pre-exists swimming; rather, as artifact, language comes into existence through language activity. By virtue of the medium language, individuals are forming their activities to each other and to themselves in interrelated movements – music or dance could be further suitable metaphorical images to exemplify this conceptualization.

Language-as-medium in this sense is the enabling and constraining element wherein thinking occurs for its social articulation (Bertau, 2014a). The basic idea of the medium-as-element contradicts both the tool and envelope metaphors in a sharp way, since these metaphors reduce language to a discrete entity at the free disposal of an agent and belong to what is known as substance ontology.

A substance ontological view prioritizes entities over processes. It is characteristic to Western thinking and the way of “describing reality as an assembly of static individuals whose dynamic features are either taken to be mere appearances or ontologically secondary and derivative” (Seibt, 2018). Movement is here a feature of entities, but not their way of being, it needs thus to be explained (Schürmann, 2006). In contrast, process ontology (or philosophy) postulates that processes are primary and that entities are formed through processes. Processes give raise to, they *form* entities as certain ones, they lead to specific substances. This means that individual entities cannot be located outside of a process, rather they are coming into being by this process (Bertau, 2016). In process ontology, the hierarchy is reversed and the entanglement of processes and entities is assumed. Movement is thus a way of being and considered as principally happening. What needs to be explained are any forms of persistence, or structure, within the movement’s flow (Schürmann, 2006). Our approach to thinking as social and embodied is based in process ontology. Insisting on the process while keeping structuring moments, we seek to understand how thinking occurs, i.e., how it develops into what we call and can delineate as “an idea” privileging processes over entities. For this reason, we explicitly opt for the metaphor of language-as-medium understood as an element (Bertau, 2014a).

*Language as medium in the sense of element* originates in Humboldt’s language philosophy viewing language as activity, as “doingness” (von Humboldt, 1999; Seifrid, 2005; Bertau, 2014b). This specific kind of activity is instrumental to thinking, but with two key differences with regard to the tool idea. First, thinking occurs for an agent related to a listening and replying other; second, language has a clear formative effect to thinking – language is the “formative organ of thought” (von Humboldt, 1999, p. 54). Humboldt’s “formative organ for thought” alters and (re-)organizes psychological processes. The partner is needed to fulfill and complete the specific forms as co-constructed and co-developed formation. For Humboldt, idea formation resides in articulating an idea through speaking to a listening and replying other. The uttered, i.e., formed languaged idea, is then reverberated back by the listener’s understanding and reply to the thinking agent’s own understanding. In this circling movement, language comes to be the medium wherein the idea comes to exist as understandable to oneself and sharable with others. On the grounds of Vygotsky’s notion of interiorization, the self-other movement can be applied to oneself thus resulting in a self-self movement (Bertau and Karsten, 2018). Since we view this reversing of the direction of address to be more important and also conceptually more specific than the location of the process (Bertau and Karsten, 2018), we prefer to speak of “self-addressed forms of speech” instead of the Vygotskian term “inner speech.”

We use the term “language activity” to signify language in this performative, immanent sense that privileges process over product (Bertau, 2011, 2014a). Interrelated with language activity, thinking has hence itself a social-dialogic (Bertau, 1999; Larrain and Haye, 2014) and concretely embodied quality – the latter qualifier specifically means *formed, shaped*. Therefore, we view semiotic means crucial to inner speech mediation (Vygotsky, 1987) as performed, situated, and embodied; language activity is this situated, embodied semiotic means, it is performed with others and for others in space and time.

Importantly, the characteristic quality of language activity includes not only dialogicality owed to the social site and addressee-orientation of the activity (“others in space and time”); it extends to its forms, or better *formations*. Speaking of the performative quality of language activity, we hence explicitly conceive language activity as per-formed activity. The performative quality thus indicates the social-dialogical unfolding of language activity with others in time and space *as well as* the forms this unfolding takes, including their modality or better: multimodality as, e.g., speech-and-gesture-gestalts. Then, dialogicality *and* formations both contribute to the social and embodied quality of mental acts; in other words, the dialogic and formative quality of language activity is kept in thinking processes. It is not converted into a complex structural string of elements or into abstracted propositions. It keeps the dialogic qualities and “evaluative accents” inherent to spoken words, i.e., to *formed* utterances (Vološinov, 1986). Language does not cease to be social-dialogical when used for the psychological individual sphere. Similarly, thinking happens for a person who, while thinking, does not cease to be that whole, engaged, affected, and embodied social being. Ideas come then into being through processes saturated with and informed by the dialogical and formative movements of language activity.

## Studying Idea Formation

For obvious reasons, it is not easy to study such complex socio-psychological processes empirically. Idea formation often happens within moments of time and is to a large extent a silent process; many ideas are never uttered aloud to oneself or to others, nor do they become written down or presented in any other perceivable form. We see the following requirements to the study of idea formation. First, a qualitative approach is needed that studies the process in a (near to) real-life situation in order to not curtail the social complexity of idea formation; second, it is necessary to work with a complex idea articulated over a larger stretch of time, so that idea formation is slowed down; a micro-developmental approach complies with a time-sensitive study of the becoming of an idea. Thirdly, the formation process should take place in an at least partly overt, perceivable fashion, in order to provide an entry point for analysis. Lastly, the subtleties of idea formation have to be grasped by a micro-descriptive and discourse-analytic approach that allows to characterize and classify the forms that are produced in sufficient detail. In the following, we present material from a prior study on writing processes that fits these requirements (Karsten, 2014b). The short excerpts from our data below make it possible to trace the becoming of an idea through a sequence of interrelated moments.

Martin, a cognitive scientist, works on the revision of a scientific paper that he received with reviewers’ comments. In the extracts of his revision process presented here, he composes a paragraph that is intended to present a central argument in the text. However, Martin struggles with the exact articulation of this central idea for his readers and, as we will see, with the fact that he needs to clarify, i.e., articulate the idea for himself, too.

Martin’s idea formation is presented in chronological steps, where the distinction of different steps is an analytical one. It refers to qualitative changes regarding Martin’s activities – what he is doing in a specific moment – and to changes in gaze, posture, and writing tools and procedures. In terms of time, the individual steps follow each other seamlessly, there is no pause in between the sequences.

**Figure 1:**
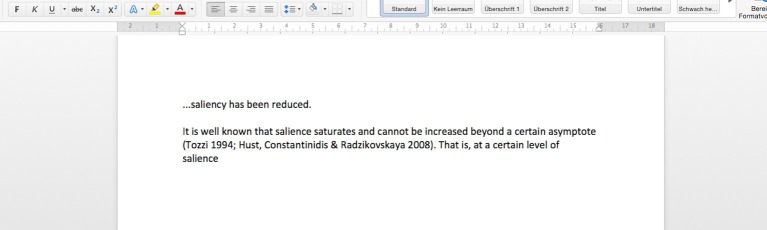
Martin writes down the beginning of a sentence (step 1).

Step 1: Martin writes down the sentence “It is well known that salience saturates and cannot be increased beyond a certain asymptote,” followed by two references. Then he continues with a new sentence in which he wants to explain the concept of salience to his readers. He stops after the words “That is, at a certain level of salience” (Figure 1).

**Figure 2:**
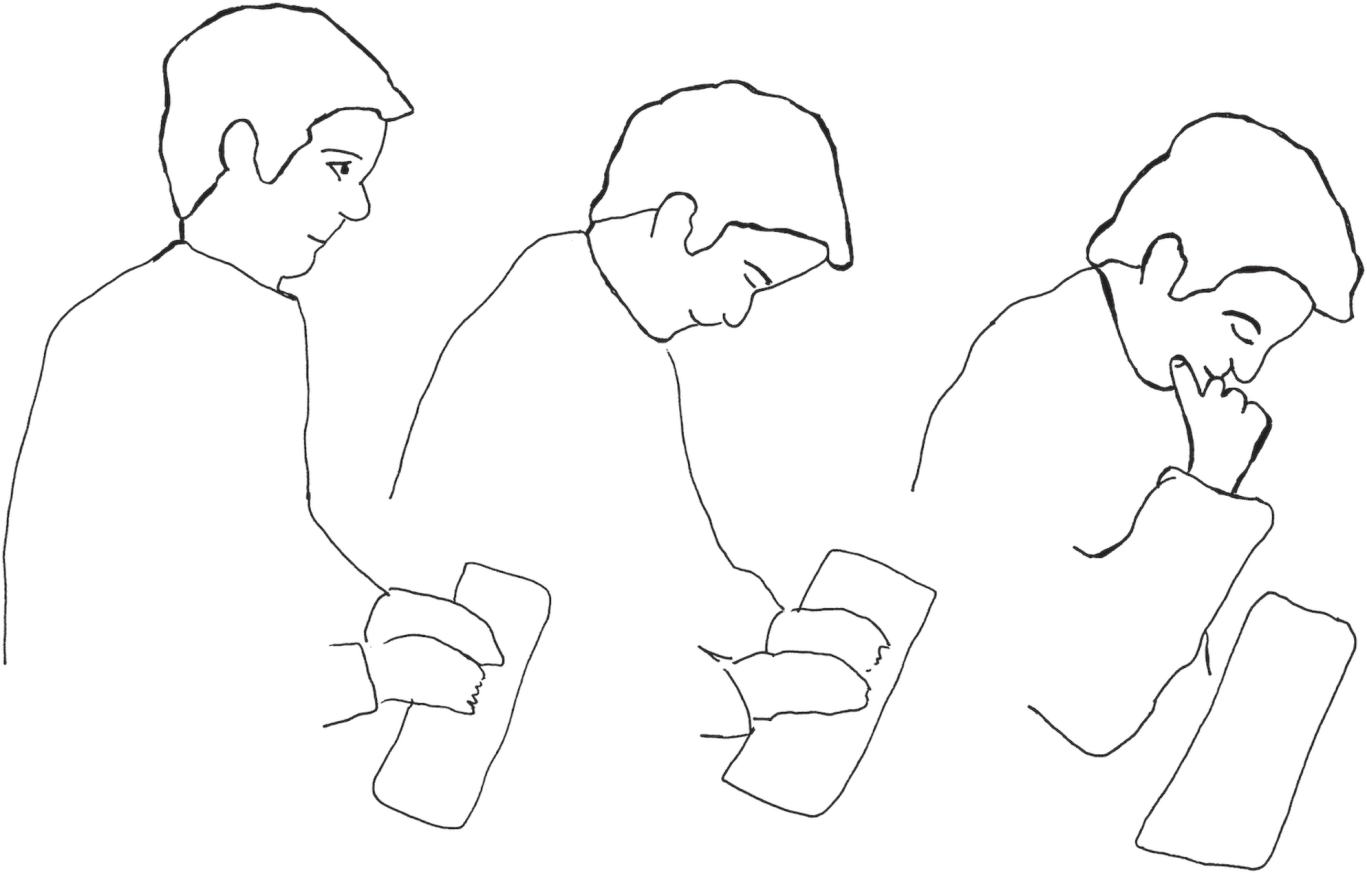
Martin pauses and thinks (step 2).

Step 2: Martin pauses after the half-sentence, with his hands still on the keyboard. His gaze first moves from the screen and stares into space, then he closes his eyes and bends his upper body. Finally, he sinks down even more and rests his chin on his hand (Figure 2).

**Figure 3:**
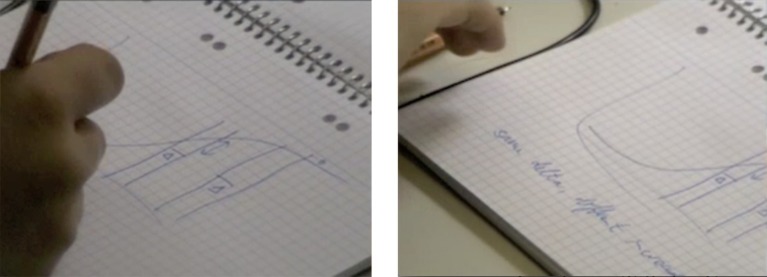
Martin draws a graph on a notebook sheet (step3).

Step 3: Immediately after, Martin turns to his notebook that lies close to the upper right corner of his keyboard. With a felt pen he draws a function curve and marks two sections of equal breadth with vertical lines and marks each with a delta symbol (Δ). One section is placed further to the left, where the curve’s gradient is steep, the other section is placed further to the right, where the line is approaching an (imagined) horizontal asymptote and the rise is smaller. Under the graphic, Martin writes a short note “same delta, different increase” (Figure 3).

**Figure 4:**
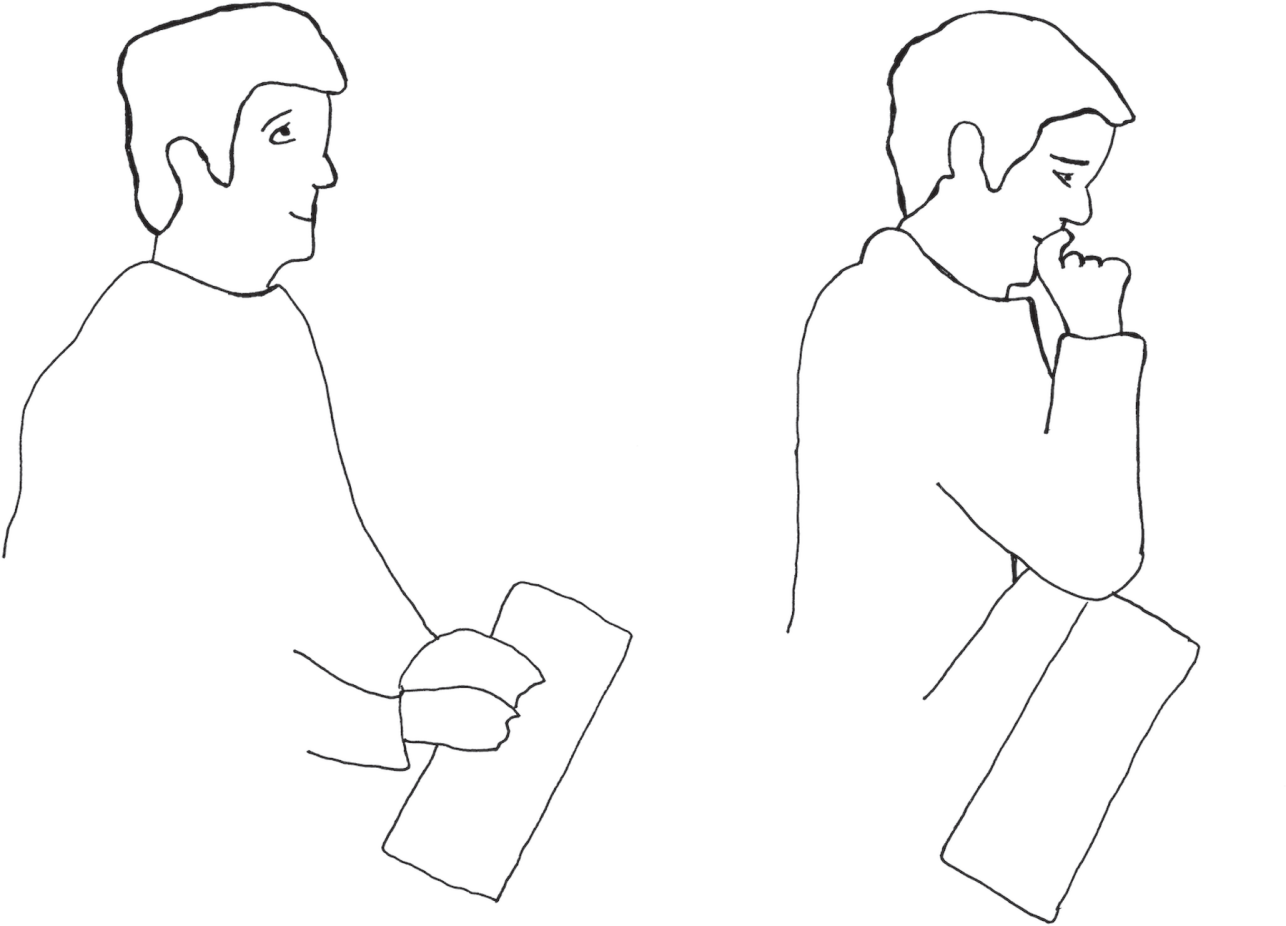
Martin sits up to continue writing and pauses again (step 4).

Step 4: Martin then turns back to his keyboard and computer, sets his fingers ready on the keyboard, and looks up into space. He rests here for some moments, then he bends down his upper body – a little less than before – and holds his right index finger to his lips in a “thinker’s pose” (Figure 4).

**Figure 5:**
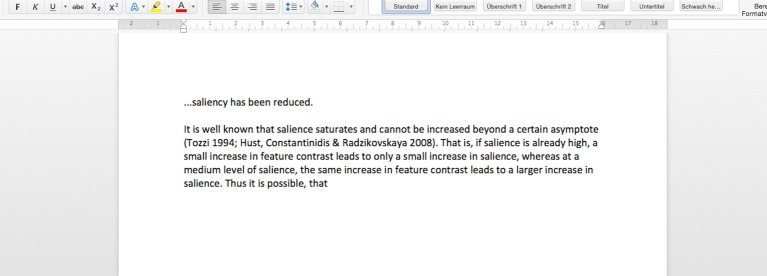
Martin finishes the sentence (step 5).

Step 5: After that, Martin quickly turns to his keyboard and screen again, deletes “at a certain level of salience” and instead continues the sentence he left unfinished before to read: “That is, if salience is already high, a small increase in feature contrast leads to only a small increase in salience, whereas at a medium level of salience, the same increase in feature contrast leads to a larger increase in salience” (Figure 5).

## Observing Condensed and Expanded Language Forms

How can we characterize and identify the peculiarities of these instances in Martin’s formation of his central idea? The Russian dialogist Jakubinskij (1979; Yakubinsky, 1997) provides a useful twofold continuum that can serve as a descriptive model to identify the exact utterance context for every instance and to provide an explanation for the associated utterance form.

Jakubinskij views human speech activity as a “manifold phenomenon” (Jakubinskij, 1979, p. 321). According to him, one can identify a broad range of speech forms in relation to various psychological and sociological factors, such as the participant constellation of a communicative situation or the socio-physical context of speaking. The complex diversity of speech forms that Jakubinskij notes resonates with our notion of speaking and thinking as social and embodied activities with an emphasis on their tangible phenomenality. With the classification schema that Jakubinskij proposes, we can analytically access the interrelation of linguistic phenomena with extra-linguistic factors.

To classify utterance contexts, Jakubinskij identifies a *dialogic* versus a *monologic* form of verbal interaction on the one hand, and he contrasts a *direct, unmediated* form of interaction with an *indirect*, *mediated* form of interaction on the other hand (Jakubinskij, 1979, pp. 323–324). These distinctions result in two analytical continua with a variety of possible intermediate forms. At the *dialogic pole* of the first continuum, utterances are highly other-dependent and expect direct response and interruptions (e.g., a conversation during family dinner or a phone call with a friend), whereas the *monologic pole* is characterized by continuous and more “self-sufficient” forms of speaking (e.g., a conference talk or a novel). At the *unmediated* or *immediate pole* of the second continuum, co-presence and thus visual-aural perception of the other is characteristic (e.g., all kinds of face-to-face interactions, also those where others function as a tacit audience or as mere overhearers and bystanders, c.f., Goffman, 1981). The *mediated pole* of the second continuum does not provide a physical co-presence, and communication is mediated by writing (e.g., a scientific article or a letter), the telephone, or, nowadays, the whole range of digital media (e.g., a chat in a messenger app or an online video tutorial). Especially with the unmediated-mediated continuum, the (multi)modality of an utterance and its resulting specific tangible phenomenality is addressed – even though Jakubinskij does not use these exact terms.

Every utterance can be classified according to both continua, e.g., as highly dialogic and highly immediate (e.g., a face-to-face dialogue between two closely acquainted persons); as highly dialogic and highly mediated (e.g., a chat on an instant messenger); as highly monologic and highly immediate (e.g., a keynote speech without the support of slides or other visualizations); as highly monologic and highly mediated (e.g., a novel); or as any intermediate form imaginable (Table 1). Note that Jakubinskij’s classification works along two organizing axes: structure of time of speech in the speaker (short duration – longer lasting duration) for continuum 1; co-presence of listening other(s) for continuum 2. Both axes together show that all speech is addressed to Other, independently of being oral or written, and whether the speaker shares the same time and space with an actual other.

**Table 1:** The four elementary forms of speech according to Jakubinskij (1979) and Yakubinsky (1997).

	Dialogic forms of speech Time: short speaking	Monologic forms of speech Time: longer lasting speaking
**Direct/unmediated** **Presence: co-present other(s)**	Short-duration speech moves in presence of other(s)	Longer talk in presence of other(s)
	Example: dinner-table conversation	Examples: conference talk, lecture, sermon
	Immediate reply expected; speech is oriented toward interruptions by listener(s)	No immediate reply expected
	Language forms: tend to be abbreviated, even fragmented	Language forms: tend to be elaborated (syntax, semantics); still have context-sensitive address forms to a listening audience (e.g., “as you know”)
**Indirect/mediated** **Presence: differed or not co-present other(s)**	Short-duration mediated speech moves, actual other(s) are either not immediately accessible or not co-present	Longer texts, often without actual other(s)
	Examples: chat in a messenger app, phone call	Examples: scientific article, novel
	Prompt reply expected; speech is oriented toward interruptions by partner(s)	No reply expected, but still anticipated, desired, imagined
	Language forms: tend to be abbreviated, but need to compensate for missing mimics and gestures	Language forms: very elaborated (syntax, semantics); context-sensitive address forms to listening audience are formalized according to genre (e.g., how to address of concurrent theory in scientific article)

According to Jakubinskij, an utterance’s degree of dialogicality and its degree of mediatedness lead to certain formal features. Dialogic forms are compositionally simpler than monologic forms (Jakubinskij, 1979, p. 334; Yakubinsky, 1997, p. 251). In dialogue, speakers have a common history both with regard to a possible shared stretch of lifetime and, most importantly, to a shared co-experience of the here-and-now micro-history and a co-construction of the given discourse. This results in shared knowledge that does not have to be uttered – the language forms are *abbreviated* and simplified syntactically (Yakubinsky, 1997, p. 256), whereas semantically they are *condensed* (Vygotsky, 1987, p. 269). Monologic forms, in turn, are more planned and focused, and their linguistic form is syntactically more interconnected (Jakubinskij, 1979, p. 324) and *expanded* (Vygotsky, 1987, p. 270), since the speaker does not know to which extent (s)he can rely on shared knowledge. Immediacy and co-presence of the other allow for modifying or substituting of the said via mimics and gestures. Thus, the speech form can be abbreviated and simplified, and “[i]n combination with speech exchange th[e] role of visual perception, indeed, remains *and sometimes prevails*” (Jakubinskij, 1979, p. 325, our emphasis). This is the reason why in our analysis we include a close look at the (multi)modality of the utterances under scrutiny. In turn, mediatedness leads to a lack of visual and aural perception of the other and thus prohibits that the different nuances of sense can be understood through means of facial expressions, gestures, intonation, and timbre (Jakubinskij, 1979, p. 326). The mediated forms of speaking must rely on “words and their combination” (Jakubinskij, 1979, p. 335) resp. their “concatenation” (Yakubinsky, 1997, p. 251) – they are thus expanded to substitute for the missing presence of the other and a shared here-and-now. Furthermore, extreme mediation like in writing has the effect that speech is fixed in its realization, and that something enduring persists (Jakubinskij, 1979, p. 335). The writer pays attention to adequacy of the utterance with regard to his or her mental states and the speech form in itself is subject to judgment (Jakubinskij, 1979, p. 334; Yakubinsky, 1997, p. 251).

With regard to our example of Martin’s writing process, step 1 can be characterized as highly monologic and highly mediated. There is only Martin present, and he must build up both his readers’ perspectives and the whole communicative situation voluntarily. There is an intended readership and the text is meant to be read, commented on, and judged by other people later, most prominently the reviewers. Yet, the production and the perception of written scientific publications are mediated and stretched over time. Adequacy with regard to content and formal characteristics of texts is key in this cultural practice. Thus, the written utterance produced in step 1 is a prototypical case of an expanded language form, due to its contextual conditions: written mode in a highly institutionalized discourse, and actual, but distant audience, that does not interfere with the text at the moment of production, but later on will do so. Interestingly, Martin seems not to be able to articulate the expanded form fully. He interrupts his writing in the middle of a sentence and continues with a very different form of activity.

Step 2 is still highly monologic according to Jakubinskij’s definition, since there is no co-present other. However, we have reason to argue that Martin lapses into inner speech (Vygotsky, 1987) or, as we prefer to call it, self-addressed speech. Physically, Martin performs a movement of withdrawal, directing his gaze away from his monitor up to almost closing his eyes and taking his hands away from the keyboard and placing one hand in front of his mouth. This is a culturally typical embodiment of self-directed thought and contemplation, a “thinker’s pose.” No present or distant others are meant to take part in this activity. In fact, Martin performs the turning-away from any others in a very strongly marked embodied fashion; he turns away from his other-directed text on the computer screen and thus withdraws himself from any interlocutor in the true sense of the word. On the second continuum, step 2 can be classified as highly unmediated. No overt language activity and no perceivable motion at all, is noticeable. The forms of Martin’s supposed covered language activity cannot be inferred simply. Thus, we will return to this instant in the next section, in order to further discuss what possible language forms are at work and what their status for idea formation is.

Step 3 is, again, highly monologic. The writing scene and the artifacts used (paper notebook and pen) implicate a genuinely monologic setting. There are still no co-present others, but there also seems to be no intended audience either. This does not mean that the Martin’s activity is un-addressed, but rather, that it is again self-addressed. This is not the official document that Martin will feed back into the review process after completion, but a personal sketch. It is not designated for a reaction or response. The third step is mediated both graphically and by writing. Aptly, bodily forms (gestures, facial expressions) do not feature particularly in this third step. In contrast to step 1, the linguistic forms used, “same delta, different increase” are highly condensed and “predicative.” Predicativity, according to Vygotsky (1987, pp. 267–268, in his interpretation of Jakubinskij), is a syntactic feature of inner speech that can also be found in external dialogic situations, when the subject of the interaction is known to all interlocutors. Predicativity is thus one feature of dialogic and immediate forms of speech, feeding into their abbreviated character. This leads to the impression that without explanation, the short “verse” Martin jots down is not understandable to others. For Martin, however, it seems to be crucial for his idea formation. Here, mediation is used in a way that contrasts with how it is used in step 1, namely in combination with a self-addressed layout. Therefore, the language forms that Martin produces are different from those in step 1: not extended and objectified, but idiosyncratic and condensed.

Step 4 is interesting because it strikingly mirrors Martin’s body movements in step 2. The analysis in terms of both continua is the same: this is a highly monologic form, but in the self-addressed sense, and a highly unmediated form as well. Again, this “thinker’s pose” needs to be scrutinized further, because of its culturally marked covert quality and its gesture of withdrawal from others. With regard to the becoming of Martin’s idea, it is interesting to note that Martin sits back at what seems to be his typical writing pose at first. But then his body posture changes from the more expressive mode to a contemplative mode. He seems not to be able to actually articulate his idea in a written, other-addressed, and expanded form yet.

Finally, in step 5, this stage is reached, and Martin continues the sentence he was not able to articulate minutes before.

Looking at the language forms that Martin produces, an interesting aspect becomes visible, which we labeled elsewhere as the ebbing and advancing of language (Bertau, 2008), more precisely, of the linguistic factors. According to both theoretical considerations following Jakubinskij and to what we will further show in our analysis, this movement observable in language activities is correlated to the speaker’s bodily activities. In the case of linguistic factors ebbing, i.e., becoming less developed, articulated, and elaborated, the speaker’s body (intonation, mimic, gestures) acts as modulator of the uttered meaning-forms and sometimes even replaces them, as in the case of Martin’s “thinker’s pose.” In the reversed case of advancing linguistic factors, the body recedes to the point of becoming invisible, not present anymore. The punctuation system in alphabetic writing can be viewed as supplying a kind of substitute for intonation (e.g., ?!) and speaking rhythm (e.g., ;,), supported in this by all kinds of layout forms. In this way, the phenomenality of language activity shows a constant oscillation, a transitional movement between condensed and expanded forms that are related to the presence of an Other and to the time amount given to the utterance.

## Varying Grades of Objectification Depending on the Constellation of Addressivity

The observed pulsating movement between condensed and expanded language forms and the related ebbing and advancing of bodily forms like posture, gesture, facial expression, or intonation is set in motion by varying extra-linguistic factors. These factors can be partly specified by Jakubinskij’s continua of dialogicality-monologicality and mediatedness-unmediatedness. But the pulsating movement also seems to be due to how much the speaking, writing, or thinking is self-addressed or other-addressed. In the following, we will investigate how the exact addressivity constellation of a given utterance co-influences a certain language form. We will further discuss how this interaction between addressivity constellation and language forms is related to a certain grade of objectification in the process of idea formation.

**Figure 6:**
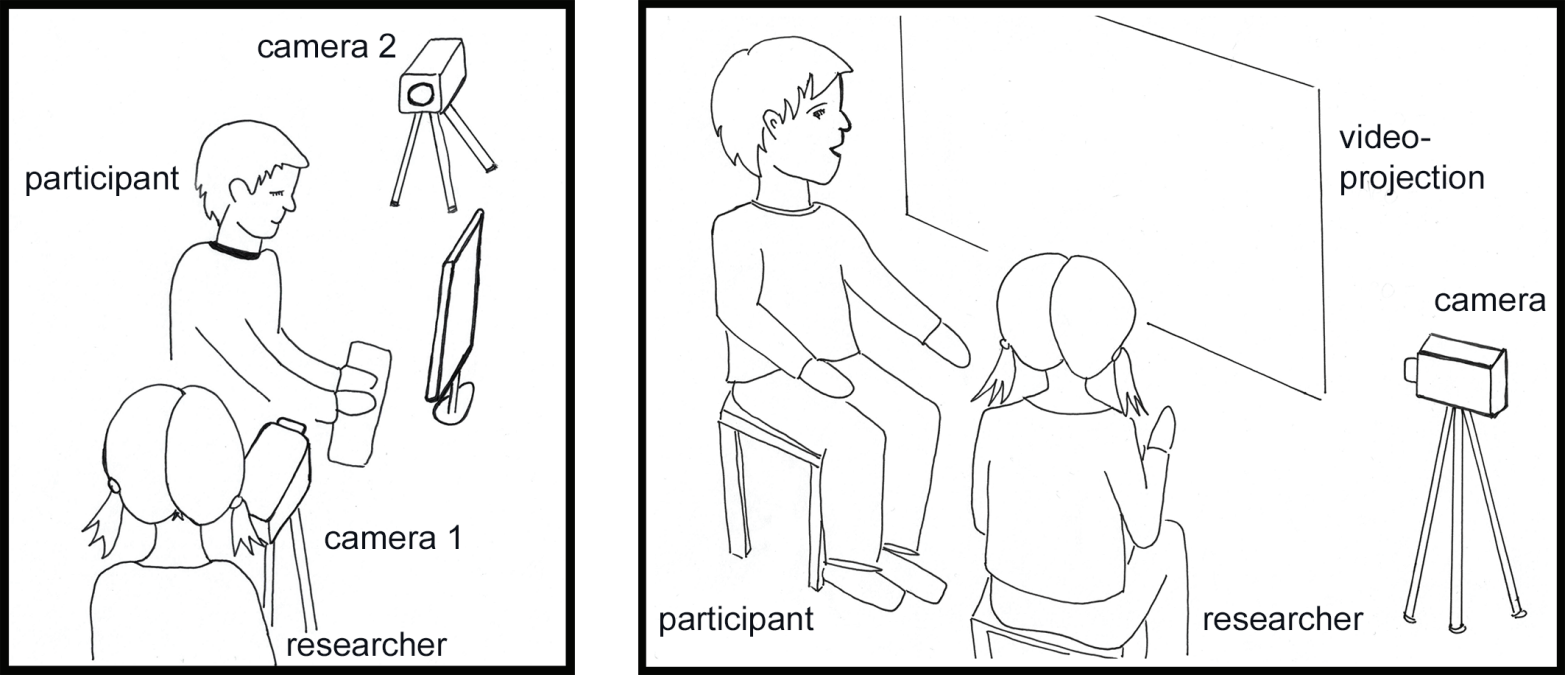
Writing process situation and video-confrontation setting.

For this purpose, we will leave the temporal sequence of Martin’s writing process at this point and turn to the ebbing and advancing moments of Martin’s idea formation in the interplay of changing addressivity constellations. In the context of the original study, Martin’s writing process was re-situated in a video-based interview, a video-confrontation. The setting is illustrated in Figure 6. It allowed for a video-based dialogical retrospection of Martin’s writing process by Martin himself together with the researcher-interviewer. For this purpose, the researcher presented parts of the video to Martin several weeks after the writing process took place. The interview format was semi-structured and the questions focused on what Martin saw himself doing on video, what his thoughts and intentions were, and what explanations he had about what he was doing there. Martin’s reconstructions were acknowledged, further explored, discussed, and sometimes called into question. We have argued in previous publications that Martin reflected and re-presented his “inner” (i.e., un-vocalized, silent) dialogues during writing in the subsequent interview setting, and this was marked by a differing basic addressivity constellation with the researcher as a co-present person and Martin’s main addressee (Karsten, 2014a,b; Bertau and Karsten, 2018).

The first transcript from the video-confrontation interview is a scene, where Martin (M) renders a first rough description of his central idea, the “argument with the curve” (line 4243) to the interviewer Andrea (A).

### Transcript 1: The argument with the curve

**Table:** 

4227 M:	das argument ist sozusagen^1^ *the argument is so to speak*
4228	naja’ *well*
4229	die leute sagen in der einen aufgabe gibts die effekte *people say in the one task the effects do (not) exist* kategorisch nicht *categorically not* (...)
4237 A:	aha *uhum*
4238 M:	jetzt [könnte es doch der fall sein]_1_ *now it could be the case*
4239	[((erhobener zeigefinger))]_1_ *holds up index finger*
4240	[dass sie vielleicht kleiner sind]_2_ *that they maybe are smaller*
4241	[((bewegt eine hand nach unten))]_2_ *moves one hand downwards*
4242	(---)
4243	und dann kann ich dieses [argument]_1_ mit der kurve [machen]_2_ *and then I can (make) this argument with the curve make*
4244	[((zeigt zur projektion))]_1_ *points towards video-projection* [((bogenförmige bewegung))]_2_ *bow-shaped movement* (See Figure 7)

**Figure 7:**
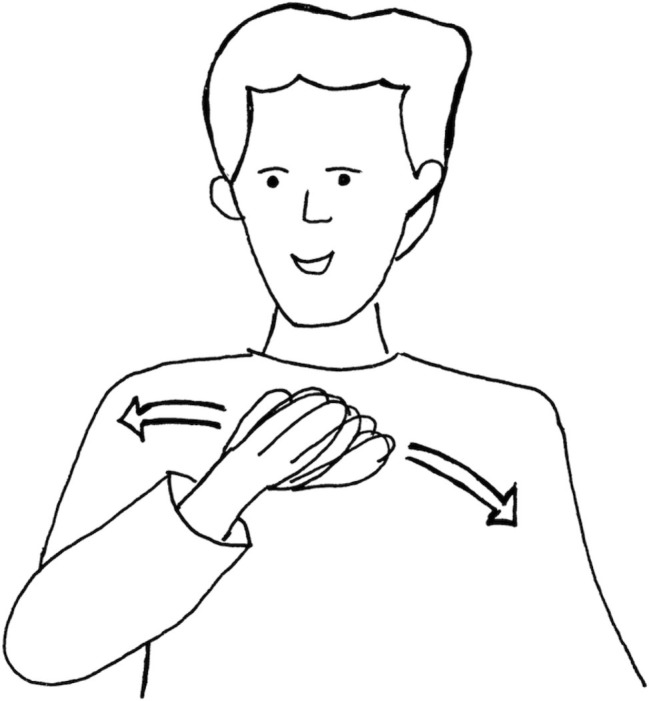
Bow-shaped gesture.

In this first transcript, there are several formations in relation to Martin’s central idea, performed in different addressivity constellations. The *first formation* is performed as enacted dialogue between “people” (line 4229) and Martin, ranging from line 4228 to line 4241. The enacted dialogue is framed by a comment to the interviewer “the argument is so to speak” (line 4227), which sets the imaginary scene for the dialogue. We can assume that “people” are either Martin’s reviewers, their respective work groups, or Martin’s research community in general including all the groups at different universities that work on similar problems as Martin and his group do. Martin first gives their position “in the one task the effects do (not) exist, categorically not” (line 4229) – the first communicative move in the enacted dialogue. Then he immediately contrasts it with his own objecting position, which he presents in the form of direct speech, accompanied with a number of gestures: “now it could be the case that they maybe are smaller” (lines 4238, 4240). This is the second move to the enacted dialogue. Martin’s raised index finger in line 4239, a culturally typical conventionalized and thus symbolic gesture, marks this move as his own personal contribution to the scientific argument between contrasting approaches. It indexes the request to speak or a kind of “veto” in a multi-party discussion.

Martin then leaves the enacted dialogue and comments to the interviewer: “and then I can make this argument with the curve” (adjusted translation, line 4243). Here, we have the *second formation* in a different addressivity constellation: a denomination of the idea as “argument with the curve” addressed to the interviewer. The semantically condensed form resembles a sort of headline or title to three instances: (1) to the whole idea formation process during Martin’s writing process that Andrea and Martin are watching; (2) to the textual section Martin composes during this stretch of video (c.f., Figures 1, 5); and (3) to the third, not yet performed move in the enacted dialogue just discussed – Martin’s paper as an elaboration and justification of his “veto” (second move) to the others’ position (first move).

Finally, the *third formation* in relation to Martin’s central idea is his gesture in line 4244, which is rendered in Figure 7. Martin’s hand movement indexes the form of the curve and is a twin form to the graphic on Martin’s notebook in Figure 3. The gesture is not symbolic, but iconic. By means of its phenomenal similarity, it functions thus as a chain between the notebook graphic and the headline formulation “argument with the curve.”

In terms of addressivity constellations, the first formation enacts an imagined constellation with the researcher as witnessing audience. The second formation is directly addressed to the interviewer, as is the third formation, which unfolds and re-enacts the past representation on the notebook for her. Within these addressivity constellations, the idea is articulated in various ways. Characteristically, the oral and gestural formations embedded in the different dialogically organized constellations do not become fully extended in contrast to the final spelled-out written argument (step 5, c.f., Figure 5). They are rather condensed forms, marked by what Vygotsky (1987, p. 277) called “influence of sense,” both in the literal meaning of infusion and in the common meaning of impact.

The next transcript sheds light on the idea “behind” the “argument with the curve.” It clarifies what Martin was trying to articulate in his unfinished sentence in step 1: “and I want to tell the people that do not have the concept: what does saturating actually mean” (adjusted translation, lines 4711–4712). The clarification occurs in the subsequent interview setting as an intertwinement of addressivity constellations so that the utterances are working and valid for several addressees.

### Transcript 2: And saturating means that…

**Table:** 

4708 M:	das find ich jetzt auch interessant *that I find now also interesting*
4709	also ich ich weiß was saturieren ist *well I I know what saturating is*
4710	ich hab n kon ich hab n mentales konzept dazu *I have a con I have a mental concept (related) to it*
4711	°h und i ich möchte aber den leuten sagen die das konzept nicht haben *°h and I I want however tell the people that do not (have) the concept have*
4712	was heißt eigentlich saturieren *what means (actually) saturating*
4713	[=stopp] *=stop*
4714	[((hebt zeigefinger))] *rises index finger*
4715 A:	((hält film an)) *stops video*
4716 M:	und saturieren heißt dass ähm *and saturating means that uhm*
4717	[wenn du hier (--) eine bestimmte strecke nach rechts gehst *if you here (--) (go) a certain stretch to the right go* hast du n GROßen gewinn *you have a GREAT gain*
4718	und wenn du hier ne strecke die gleiche strecke nach rechts gehst *and if you here (go) a stretch the same stretch to the right go* hast du einen kleinen gewinn] *you have a small gain*
4719	[((steht während seines turns auf, zeigt an projektion und setzt sich wieder))] *gets up during his turn, points at video-projection and sits down again*
4720 A:	mhm *uhum*
4721 M:	das heißt saturieren *that means saturating*

While Martin and the interviewer are watching step 1 (the unfinished sentence, c.f., Figure 1) and 2 (the first thinker’s pose, c.f., Figure 2) of Martin’s idea formation process, Martin says that he indeed knows what saturation means (line 4709), and that he was going to explain the idea of saturation to those readers who do not know this (lines 4711–4712). However, we can assume that he was not able to fully articulate this idea yet, because he interrupted his composing process (step 1). We further assume that he tried to articulate the idea, his “mental concept” in his own words (line 4710), silently while sitting and thinking (step 2). According to Martin’s reconstruction, step 2 is indeed the attempt to find an articulation for his readers. Martin again renders his aims during writing in the form of an *enacted dialogue* with the interviewer as audience. To determine the addressivity constellation, note that Martin does not use indirect speech with a relative clause (≈ and I want to tell the people that do not have the concept *what saturating actually means*), but direct speech. This results in the formulation of a direct rhetorical question, addressed to his imagined readers: “what does saturating actually mean?” (adjusted translation, line 4712). As we know, this question, which might have been there during Martin’s thinking in step 2, is not answered in the text immediately. Instead steps 3 (graphic on the notebook, c.f., Figure 3) to 4 (second thinker’s pose, c.f., Figure 4) follow before Martin seems to be able to articulate the answer in his text (step 5, c.f., Figure 5).

In line 4713, Martin asks Andrea to stop the video – the image shows his graphic sketch of the function curve (step 3). Interestingly, he simultaneously raises his index finger (line 4714), calling for his interlocutor’s attention in the same way as he did moments before in the context of the projected dialogue with the addressees of his text (c.f., analysis of transcript 1). Martin goes on to say: “and saturating means that” (line 4716). Instead of answering the rhetorical question for the intended readership, Martin transcends the two contrasting addressivity constellations by giving the answer to the co-present interviewer: “and saturating means that uhm if you here (--) (go) a certain stretch to the right go you have a GREAT gain, and if you here (go) a stretch the same stretch to the right go you have a small gain” (lines 4716–4718). Evidence for this interpretation are the deictics “here” in line 4717 and 4718 together with Martin’s getting up and pointing to the two sections of the curve at the video-projection (line 4719). His finishing phrase “that does saturation mean” (adjusted translation, line 4721) is valid for several addressivity constellations. It is an affirmation addressed to the interviewer, a concluding answer addressed to his imagined readers in the enacted dialogue and maybe a reflection of his thinking during step 2 (first thinker’s pose, Figure 2).

The next analysis clarifies this supposedly occurring self-addressed phase during Martin’s writing (step 2). The video-confrontation interview allows a glimpse into the idea formation process that takes place in silence. Prior to the following episode, Martin related that his thinking is not completely verbal and that often a translation between non-verbal thoughts into words needs to happen. Andrea asks Martin for a retrospective introspection: “what is that kind of thinking like, if it is not with words?” (adjusted translation, line 4868).

### Transcript 3: Almost with moving

**Table:** 

4868 A:	wie wie ist dieses denken wenns nicht mit worten ist dann *how how is that kind of thinking if it (is) not with words is then*
4869	also *well*
4870	so wenn du dem irgendwie nachspürst *so if you (into it) somehow feel into it*
4871	was da passiert wenn du da sitzt und denkst *what there happens when you sit there and think*
4872 M:	(12.0)
4873	aʔ
4874	ist interessant was das für ne modalität ist *(it) is interesting what kind of modality that is*
4875	das_s fast mit bewegen *that is almost with moving*
4876 A:	mhm
4877 M:	also das ist fast also *well that is almost well*
4878	((stellt teetasse ab)) *puts down tea cup*
4879-	zu wissen dass [des]_1_ und [des]_2_
4883	*to know that this and this* [((klammergeste mit linker hand))]_1_ *bracket-formed gesture with left hand* [((klammergeste mit rechter hand))]_2_ *bracket-formed gesture with right hand* einmal [sowas]_3_ und einmal [sowas]_4_ macht *(makes) one time such a thing and the other time such a thing makes* [((geste weiter oben mit linker hand, größere spanne))]_3_ *gesture further up with left hand, greater span* [((geste weiter oben mit re. hand, kleinere spanne))]_4_ *gesture further up with right hand, smaller span* (See Figure 8)
4884 A:	mhm
4885 M:	also *well*
4886	s s (-) SO is das bloß ohne s zu machen *l l (-) LIKE THAT is that only without doing it*

**Figure 8:**
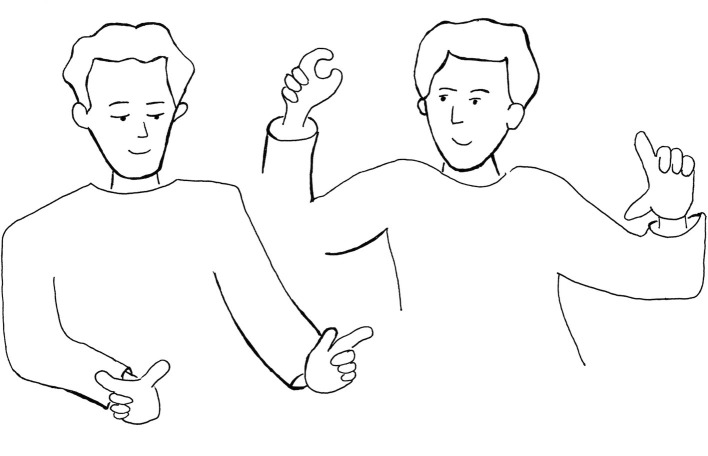
This and this makes that and that.

The question the interviewer asks in line 4868 explicitly refers to Martin’s first thinker’s pose (step 2): “if you somehow feel into it what happens, when you sit there and think” (adjusted translation, lines 4870–4871). Notably, Martin takes his time to answer (line 4872). The 12 s of silence are indications that Martin re-lives his thinking process. His answer seems to be somewhat surprising to himself as the glottal stop particle “aʔ” (line 4873) suggests, together with the comment: “it is interesting what kind of modality that is” (adjusted translation, line 4874). Martin concludes that his thinking process is highly embodied, “almost with moving” (line 4875). At this point, Martin describes exactly what we have deduced from Jakubinskij’s theory previously: an almost total ebbing of linguistic factors and the speaker’s (or thinker’s) body movement as carrier of the not-yet verbalizable meaning.

In the next step, this not-yet-verbal form gets re-enacted for the interviewer (lines 4879–4883 and Figure 8). Interestingly, there is a verbal part of the reconstruction, but it is highly deictic and almost does without denominations: “well that is almost as if to know that *this* and *this* results in something *like this on the one hand* and something *like that on the other hand*” (adjusted translation, lines 4879–4883). This husk-like verbal form is accompanied (or rather: completed) by a gesture-movement-complex (Figure 8). Martin first performs a bracket-formed gesture with his left hand at the first “this,” then another bracket-formed gesture of the same size and form with his right hand at the second “this.” Then he moves his left hand a little further up, rotates it, and enlarges the span between his index and thumb with “like this.” Finally, he also rotates his right hand, moves it even higher than his left hand, and lessens the span between index and thumb with “like that.”

The gestural forms and their locations in front of Martin’s upper body are clearly twin forms of the delta distances in the notebook graphic and the respective risings of the curve within these deltas (see Figure 3). Like the bow-shaped gesture analyzed above, these forms are not conventionalized symbolic gestures, but iconic ones, resembling their twin forms on Martin’s notebook in size and shape. Further, Martin’s re-enactment of his thinking process is exactly this: a re-enactment. It is not to be mistaken for the “original” thinking; it is a form addressed and exteriorized for the interviewer. Martin even names this fact: “it is like that, *only without doing it*” (adjusted translation, line 4886) and “well that is *almost* well” (line 4877). However, from the formal resemblance between the re-enactment of the thinking in step 2 and the graphic representation in step 3, we can infer that Martin’s bodily felt movement has a similar form as well. Another twin form to this idea that we have identified is Martin’s condensed verse “same delta, different increase” that he jots down under the graphic in step 3 (Figure 3). In the re-enactment, we can identify further resemblances in form and meaning: the two first bracket gestures actually resonate with “same delta,” whereas the two following rotated bracket gestures emblematize the “different increase.”

It is at this stage of the analysis that we have to point to a theoretical move we want to make in order to understand how ideas come into being in various grades of objectification and publicness. The linguist Jakubinskij conceives of the distinguishing criteria and the affiliated more condensed or expanded language forms we have used in our analysis (and of their respective gestural and intonational substitutes or modulators) exclusively for the pragmatic field of social language usages. Our analysis of addressivity constellations showed, however, that it is crucial for the way the idea is formed *who the addressee is* as well as *how this real or imagined other (or self!) is addressed*. The theoretical move we see as key to understand how ideas come into being is thus to translate *all* of Jakubinskij’s differentiations and possible language and bodily forms occurring in the social pragmatic field – and thus also the specific (multi)modality of an utterance – to self-addressed speech. We do this in explicit contrast to Vygotsky (1987), who – as we have given to understand – also applies Jakubinskij’s ideas to his research object inner speech (we reformulated as self-addressed speech). As opposed to our analytical and theoretical suggestion, Vygotsky translates *only one* of these forms to self-addressed speech, namely the highly abbreviated and condensed form known from immediate dialogic interaction that he labels “absolute predicativity” (Vygotsky, 1987, p. 267). Our analysis shows, however, that there are in fact many possible forms for self-addressed speech. In Martin’s case, this is most visible in the difference between (and resemblance of!) the non-verbal felt body movement (inferred from its later re-enacted form), the graphic sketch and the verbal, yet highly condensed verse “same delta, different increase.” All three forms are interrelated, and they are also crucially related to Martin’s final maximally expanded formation to his idea in step 5, when he articulates the idea for his readers in the form of the sentence: “That is, if salience is already high, a small increase in feature contrast leads to only a small increase in salience, whereas at a medium level of salience, the same increase in feature contrast leads to a larger increase in salience” (Figure 5).

How can we understand which processes take place here? Our theoretical access is the notion of objectification. The term objectification denotes a process with two interrelated aspects. First, it is a process *generating an object* as recognizable, delineated entity; second, it is a process *leading to and tying into objectivity* that pertains to common, social, or trans-individual activity types – language activity par excellence. Their meeting point is language in the sense of the embodied-performative and symbolic-conventionalized activity put forth in the Humboldtian tradition: it creates objects in a certain sense and objectifies, also in a certain sense. This understanding rests upon interconnected ideas about language, thinking, and consciousness that start in Humboldt and are taken up by Vološinov and Vygotsky (Bertau, 2014b). For these thinkers, objectification is a process of transformation which describes a movement between subjectivity and objectivity, between the individual and the social, public sphere. Within this movement, ideas are generated, thoughts are articulated and become understandable, i.e., sharable meaning-forms are shaped out. Stating the formative function of language for thought, Humboldt refers to articulation as a core moment in the thinking process, a moment that generates discernible entities (*articulus* being the small joint within a moving whole). As mentioned previously (section “Theoretical Framework”), Humboldt does not only conceptualize the formative function of language for thought, he assigns a core role to the listening-replying other in this process. In this way, otherness grounds the process of objectification.

Looking first at how an *object* is generated, a trans-formation is conceived that leads from the idiosyncratic, dense, and fluid sense with highly unstable and moving, emerging-dissolving fragmented forms to the societal stabilized meaning-forms. Abbreviated language forms such as Martin’s self-addressed verse “same delta, different increase” (Figure 3) or his deictic dialogic “this and this makes that and that”-form (Figure 8) can be located in-between the idiosyncratic and the societal stabilized forms. The contrast between sense and meaning is Vygotsky’s (1987) framework to explain the dynamics occurring between thought and word in inner speech toward external speech, it rests itself on seminal ideas of Paulhan (1928; Bertau, 2014b). Vygotsky uses the term objectification alongside with materialization (Vygotsky, 1987, p. 257, 280): thoughts become tangible, they get a materialized form in language activity (oral or written) through the mediation of inner speech. Vološinov views the occurring materialization more radically as pertaining to inner speech *already*, for an “incarnation” is needed to consciousness – incarnation being nothing but objectification: “*Outside objectification, outside embodiment in some particular material* (the material of gesture, inner word, cry), *consciousness is a fiction*” (Vološinov, 1986, p. 90). The short list Vološinov provides in brackets is a precious complement to Vygotsky’s (Paulhan’s) inner sphere of sense being explicitly located outside of any language forms as “pure meaning.” This inclusion can be linked to the broad and explicitly multimodal notion of language Vološinov advocates for, taking up his teacher Jakubinskij (Bertau, 2008).

In the previous section, we have illustrated, how Jakubinskij’s notion of language puts forth language forms (speech forms) as dynamic and anchored in life; they reach into the non-verbal dimension of any language activity thus providing the tangible interface between language and its actual reality. We have shown how the speaking body is not disregarded, its speaking-listening postures, gestures, intonations and inflections, its gazes and rhythms. As Jakubinskij says, in oral-dialogic communication, meaning is modified by the body (Jakubinskij, 1979, p. 325). So the Soviet language thinkers start with an embodied notion of language, and for Vološinov, this reaches into thinking, inner speech, consciousness.

Concerning the second side, the *objectivity of language* is a highly specific type of objectivity for it is bendable toward individual diversity and toward objectivity or, as von Humboldt (1999) puts it, the objective. Following Humboldt, this comes from the fact that language’s first purpose is to communicate something to the fellow societal partner. In Martin’s case, this is his readers, those who do not know what saturation is and those he wants to convince with his “argument with the curve.” Verbal communication is necessarily pregnant with individuals and their commonality, which they have agreed upon, conventionalized and that transcends each of them for the sake of communion-communication. Thus language needs to be, and is for Humboldt, subjective *and* objective, where the objective does precisely not reach a detachable and absolute value (Di Cesare, 1996) but stays with the individuals who need to articulate their uniqueness to each other *as* socialized individuals. In our example, Martin’s idea never fully “leaves” the fundamental dialogue it is meant for, i.e., the extremely mediated and monologic, yet explicitly other-addressed setting, where Martin objects the other researchers’ argument by his “veto” (c.f., the analysis of transcript 1). His search for a communicable meaning-form is not a search for an abstract, “true” envelope for his idea, but a trajectory toward a dialogically functional, shared objectification. The power of language resides in holding the subjective within the objective and allowing the subjective to live within the objective – in fact, to ever-replenish the objective through each language activity, which is individually unique while making use of others’ words, heard in others’ mouth.

## Intrapersonal Intertextuality: A Crucial Process in Idea Formation

It is exactly the concept of the individual-in-the-public and the subjective-in-the-objective that builds the ground for understanding idea formation as a complex intertextual process. As said, language-as-activity emphasizes the performative aspect of language and highlights the dynamics of multimodal forms and formations taking place in time and through time. The time dimension is crucial. It connects language activities and their forms to each other, forming a dialogical texture, or, as Bakhtin (1986, p. 91) put it, a dialogical chain between utterances. These connections between utterances occur within a given verbal communication between actual partners as well as in a trans-temporal way, thus relating the speech forms of speakers (same and different ones) across time. Language itself is understood as these wide-reaching dialogical relationships, echoing, questioning, re-taking, and altering each other in each specific moment of being uttered. Speaking amounts to join into and to weave oneself in this wide and dynamic net of mutually dialogically responding, language (or speech) forms (Bertau, 2014c). As we interpret it, the Bakhtinian term of intertextuality (as it was termed by Kristeva, 1980) highlights the dialogical intertwinement between embodied utterances or texts, not between people. In fact, the conceptual shift from dialogically related *speakers* to dialogically related *utterances* (then also voices) is prepared by Jakubinskij (1979) putting forth the idea of the interdependency of utterances, and completed by Bakhtin (1986) and Vološinov (1986). Shifting the conceptual focus of dialogicality from the uttering individuals to the utterances themselves is an important theoretical move that sheds clear light on the functioning of language-as-activity: it is exactly by detaching, by emancipating the utterance from a speaking body that the spoken and listened-to word gets its communicative-cognitive power; it is through this detachment that different dialogical forms and their voices can interfere and merge in one speaker in speaking *and* in thinking (Bertau, 2011; Gratier and Bertau, 2012). The concept of intertextuality encompasses the interrelated (or interdependent) forms and formations of language-as-activity that take place in and through time; in this sense, intertextuality happens for speakers as much as it is created by them as they re-enact others’ words within new addressivity constellations; they alter and saturate these words with a new, present-moment usage that receives the specificities of that moment in time. Taking the broad and dialogical notion of the Soviet language thinkers seriously, intertextual reprises will include embodied dimensions such as intonations and gestures to utterances and they will take place within and across modalities.

In our material, we find such intertextual reprises that conform with our reading of the concept of intertextuality. There is, however, one peculiarity: all of these forms are utterances by just one speaker, Martin, but performed in different moments, with differently embodied forms and directed to different addressees. Concluding our analysis, we sample the two most striking groups of Martin’s intertextual reprises (what we have called “twin forms” until now):

Intertextual reprises, group I:

1. the title-like formulation “the argument with the curve” (transcript 1)
2. the graphic representation of the functional curve (Figure 3)
3. the bow-shaped gesture (Figure 7)

Intertextual reprises, group II:

1. the graphic representation of the deltas distances in the notebook graphic and the respective risings of the curve within these deltas (Figure 3)
2. the handwritten verse “same delta, different increase” (Figure 3)
3. the husk-like oral explanation to the interviewer: “this and this results in something like this on the one hand and something like that on the other hand” (transcript 3)
4. the sequence of gestures during Martin’s explanation to the interviewer (Figure 8)
5. the supposed felt body movement during Martin’s thinker’s pose (Figure 2, transcript 3)
6. the final sentence in the written text: “if salience is already high, a small increase in feature contrast leads to only a small increase in salience, whereas at a medium level of salience, the same increase in feature contrast leads to a larger increase in salience” (Figure 5)

Following Vološinov’s and Vygotsky’s line of thought and our analytical findings, the concept of intertextuality seems also to be suited to describe intrapersonal dialogical relations between multimodal utterances like the ones we have sampled here. Again, this is supported by the conceptual shift accomplished by the Soviet focusing on utterances rather than on speaking individuals (Bertau, 2011). Making this theoretical extension, we use the term *intrapersonal intertextuality* to grasp the intertextual reprises performed by one and the same speaker in different moments. Our proposition is that Martin’s idea needs the intrapersonal intertextual saturation throughout both reprise groups to become fully objectified (in the Humboldtian sense), articulated, and extended in the written sentence. This is supported by the following transcript, where Martin reflects the dynamics of his idea formation process.

### Transcript 4: And then I can say it

**Table:** 

4745 M:	das ist geNAU das was ich sagen wollte sozu[sagen] *that is exACTly that what I wanted to say so to speak*
4746	A: [mhm]
4747 M:	aber ich konnts vorher nicht sagen *but I could (not say) it before not say*
4748	und dann kann ichs sagen *and then I can say it*

According to Martin’s own retrospection, he needed to perform his idea throughout these consecutive formative moments to be able to articulate it in an expanded, written, and other-addressed form: “and then I can say it” (line 4748). To him, the process is indeed about *one* idea – “that is *exactly* what I wanted to say,” he affirms (line 4745). However, this idea was not fully graspable at first; it only became more explicable and more objectified through the intertextual process.

## Conclusion

The manifoldness of embodied language forms (including indexical and symbolic gestures, postures, intonations, etc.) along with their intertextual density in which Martin (and the other participants in the original study, for that matter) re-constructs and explains his thoughts and doings to the researcher during the interview is striking. Taking these forms together with the many related forms already produced “naturally” in the writing situation (see sections “Studying Idea Formation” and “Observing Condensed and Expanded Language Forms”), one can observe movements and interrelations between body movements, gestures, drawings, self-directed talk, addressed explanations, written text, and many more. Through such a multiplicity of formations, also the “the argument with the curve,” on which we focused our present analysis, develops from a fuzzy, condensed bodily feeling to a spelled-out intertextually saturated written definition, throughout moments, situational settings, and communicative formations. Our main conclusion from our theoretically underpinned analysis is that Martin’s idea in fact only exists in (or rather: *as*) these forms of realization. The linguistic means that are mobilized during the trajectory between these realizations ebb and advance, depending on the addressivity constellation and the grade of objectification that is reached in each moment. Looking at this formative process, we argue that thinking is social, embodied, multimodal, and dialogically organized *because* it is entangled with language. It is related to others in the social sphere, it is enacted for communicative and cognitive purposes, and it is embodied because of the various language forms it takes, which show different grades of publicness, of formal expansion, and of semantic condensation. Ideas come into being by becoming uttered and addressed to self and others in culturally and historically specific language practices, thusly made objective and public, while staying fundamentally intertwined with other forms of embodiment.

## Data Availability Statement

The datasets for this manuscript are not publicly available because the publication of full interview transcripts from qualitative doctoral dissertation studies is not common in Germany. The full German transcript from the original study is available on request to interested researchers. Requests to access the datasets should be directed to AK, andrea.karsten@upb.de.

## Ethics Statement

All names occurring in the material are pseudonyms. As per applicable institutional and national guidelines and regulations, an ethics approval was not required for the original study (Karsten, 2014a). Participants in that study gave written informed consent for research participation and publication of data and case descriptions.

## Author Contributions

AK and M-CB wrote the manuscript. AK designed and carried out the prior study, from which data were re-analyzed.

### Conflict of Interest

The authors declare that the research was conducted in the absence of any commercial or financial relationships that could be construed as a potential conflict of interest.

## Footnotes

^1^The German transcript follows the conventions of the GAT 2 transcription system (Selting et al., 2009). [] mark overlapping speech or acts, sometimes clarified by subscript numbers; (()) mark comments to the speakers’ turns, describing mainly their actions, gestures, and body movements; () mark short (-), medium (--), or long (---) pauses, or the exact length of the pause in seconds is indicated within the brackets; °h marks breathing in, capitalization marks stressed syllables. A rough English transliteration is given beneath every turn. Note that this translation is meant to render the German syntax and wording as exactly as possible. Adjusted and smoothed translations are given in the text, when the respective turn is analyzed.
